# Mitochondrial Creatine Kinase 2 (Ckmt2) as a Plasma-Based Biomarker for Evaluating Reperfusion Injury in Acute Myocardial Infarction

**DOI:** 10.3390/biomedicines12102368

**Published:** 2024-10-16

**Authors:** Alexander Lang, Daniel Oehler, Marcel Benkhoff, Yvonne Reinders, Maike Barcik, Khatereh Shahrjerdi, Madlen Kaldirim, Albert Sickmann, Lisa Dannenberg, Amin Polzin, Susanne Pfeiler, Malte Kelm, Maria Grandoch, Christian Jung, Norbert Gerdes

**Affiliations:** 1 Division of Cardiology, Pulmonology and Vascular Medicine, Medical Faculty and University Hospital, Heinrich-Heine University, 40225 Düsseldorf, Germany; lang@hhu.de (A.L.); daniel.oehler@med.uni-duesseldorf.de (D.O.); marcel.benkhoff@med.uni-duesseldorf.de (M.B.); maike.barcik@med.uni-duesseldorf.de (M.B.); khatereh.shahrjerdi@med.uni-duesseldorf.de (K.S.); madlen.kaldirim@med.uni-duesseldorf.de (M.K.); lisakristina.dannenberg@med.uni-duesseldorf.de (L.D.); amin.polzin@med.uni-duesseldorf.de (A.P.); pfeiler@hhu.de (S.P.); malte.kelm@med.uni-duesseldorf.de (M.K.); christian.jung@med.uni-duesseldorf.de (C.J.); 2 Leibniz-Institut für Analytische Wissenschaften—ISAS—e.V., 44139 Dortmund, Germany; yvonne.reinders@isas.de (Y.R.); sickmann@isas.de (A.S.); 3 Medizinisches Proteom-Center, Ruhr-Universität Bochum, 44801 Bochum, Germany; 4 Department of Chemistry, College of Physical Sciences, University of Aberdeen, Aberdeen AB24 3FX, UK; 5 Cardiovascular Research Institute Düsseldorf (CARID), Medical Faculty and University Hospital, Heinrich-Heine University, 40225 Düsseldorf, Germany; maria.grandoch@uni-duesseldorf.de; 6 Institute for Translational Pharmacology, Medical Faculty and University Hospital, Heinrich-Heine University, 40225 Düsseldorf, Germany

**Keywords:** myocardial infarction, reperfusion injury, mitochondrial damage, biomarker

## Abstract

Background/Objectives: Acute myocardial infarction (AMI), characterized by irreversible heart muscle damage and impaired cardiac function caused by myocardial ischemia, is a leading cause of global mortality. The damage associated with reperfusion, particularly mitochondrial dysfunction and reactive oxygen species (ROS) formation, has emerged as a crucial factor in the pathogenesis of cardiac diseases, leading to the recognition of mitochondrial proteins as potential markers for myocardial damage. This study aimed to identify differentially expressed proteins based on the type of cardiac injury, in particular those with and without reperfusion. Methods: Male C57Bl/6J mice were either left untreated, sham-operated, received non-reperfused AMI, or reperfused AMI. Twenty-four hours after the procedures, left ventricular (LV) function and morphological changes including infarct size were determined using echocardiography and triphenyl tetrazolium chloride (TTC) staining, respectively. In addition, plasma was isolated and subjected to untargeted mass spectrometry and, further on, the ELISA-based validation of candidate proteins. Results: We identified mitochondrial creatine kinase 2 (Ckmt2) as a differentially regulated protein in plasma of mice with reperfused but not non-reperfused AMI. Elevated levels of Ckmt2 were significantly associated with infarct size and impaired LV function following reperfused AMI, suggesting a specific involvement in reperfusion damage. Conclusions: Our study highlights the potential of plasma Ckmt2 as a biomarker for assessing reperfusion injury and its impact on cardiac function and morphology in the acute phase of MI.

## 1. Introduction

Acute myocardial infarction (AMI) is one of the most common clinical manifestations of cardiovascular disease, representing a primary cause of mortality and affecting more than 3 million people worldwide each year [1]. Myocardial ischemia is caused by a reduction in coronary blood flow and a lack of oxygen supply, resulting in irreversible damage to the heart muscle and impairment of systolic and diastolic function [2,3]. The timely restoration of blood flow to enable reperfusion is widely accepted as the most effective measure to salvage vital myocardium. However, the reperfusion of ischemic tissue itself can induce mitochondrial enlargement and the release of mitochondrial pro-apoptotic components, particularly cytochrome c, into the cytosol, where they may initiate the apoptotic cascade [4,5,6,7]. Mitochondrial reprogramming, dysfunction and damage to its ultrastructure are established hallmarks of a pathologically affected heart [8].

Current technology allows for profiling the plasma proteome and utilizing it in biomarker discovery [9]. A higher abundance of mitochondrial proteins indicates multiple cell death-associated conditions, like ischemic stroke [10], prostate cancer [11], intensive interval exercises [12] and MI [13]. This underscores its indispensable contribution to ATP synthesis and respiratory chain performance [14,15]. Mitochondrial stress and dysfunction are strongly implicated in the progression of cardiac diseases, making it an attractive target for therapeutic strategies [16].

This study aimed to identify plasma proteins associated with mitochondrial damage following cardiac ischemia and reperfusion (I/R) damage. We identified candidate proteins and tested differential expression in reperfused and non-reperfused AMI. We further evaluated mitochondrial creatine kinase 2 (Ckmt2) as a reliable and specific marker in the plasma of mice subjected to reperfused versus non-reperfused AMI, as well as in sham-operated controls. Additionally, we investigated the correlations between plasma levels of Ckmt2 and cardiac functions, such as ejection fraction.

## 2. Materials and Methods

### 2.1. Mice

Male C57BL/6J wild-type mice were purchased from Janvier Labs (Saint-Berthevin, France). All mice were 10–12 weeks of age at the beginning of the experiments and received standard chow and drinking water ad libitum. Mice were kept in climate-controlled rooms with a 12 h light/dark cycle. All animal experiments were performed according to ARRIVE (Animal Research: Reporting of In Vivo Experiments) II guidelines and approved by LANUV (North Rhine-Westphalia State Agency for Nature, Environment and Consumer Protection) in accordance with the European Convention for the Protection of Vertebrate Animals used for Experimental and other Scientific Purposes (License Approval Numbers: 81-02.04.2020.A225).

### 2.2. Induction of Reperfused AMI

After general anesthesia under ventilation at a respiratory volume of 0.2–0.25 mL and a respiratory rate of 140 breaths per minute with isoflurane (3%) and oxygenated air (30%), mice underwent either sham surgery or a 45 min occlusion of the LAD to induce AMI, as previously described [17]. Following 45 min of ischemia controlled by visual inspections and changes in the electrocardiogram with the regular monitoring of body temperature (37 °C), the occlusion was resolved and the myocardium reperfused at various time points. Animals received buprenorphine (0.1 mg/kg body weight) subcutaneously.

### 2.3. Induction of Non-Reperfused AMI

After general anesthesia with ketamine and xylazine and ventilation at a respiratory volume of 0.2–0.25 mL and a respiratory rate of 140 breaths per minute with isoflurane (3%) and oxygenated air with 30% oxygen, mice underwent permanent occlusion of the LAD to induce AMI, as previously described [18]. Successful ligation was controlled by the occurrence of a characteristic ST-elevation in the electrocardiogram. Animals received buprenorphine (0.1 mg/kg body weight) subcutaneously and were controlled for further experiments until sacrifice. Another difference to the reperfused AMI model was the immediate closure of the chest after the control of the successful ligation of the LAD.

### 2.4. Plasma Protein Analysis via ELISA

CKMT2 measurements were conducted using murine plasma samples collected one day after the ischemia-reperfusion (I/R) operation. Plasma specimens were isolated by centrifuging EDTA blood for 15 min at 1000× *g* and promptly frozen and stored at −80 °C until analysis, with a maximum storage duration of six months before analysis. CKMT2 quantification was carried out following the manufacturer’s instructions using a CKMT2 enzyme-linked immunosorbent assay (ELISA) kit (Abbexa, Cambridge, UK, abx529771).

### 2.5. Echocardiography

A Fujifilm Visualsonics Vevo 3100 Ultra-High-Frequency Imaging Platform (18–38 MHz linear array micro scan transducer; Visual Sonics, Toronto, ON, Canada) was used to acquire cardiac images and perform all functional analyses. Mice were anesthetized with 1.5–2% isoflurane and placed on a heated, bench-mounted adjustable rail system with constant monitoring of their electrocardiogram, respiratory rate, heart rate and body temperature. A chemical hair remover was used to scrape off the fur from the chest of mice to improve cardiac image quality. Imaging was performed along the parasternal long axis. Cardiac parameters such as the LV chamber volume in EDV and ESV were measured in B-Mode using the Vevo LV-Trace function and strain. SV, EF and cardiac output (CO) were calculated with EDV and ESV. Imaging was performed on untreated mice prior to surgery and at 24 h post-surgery.

### 2.6. Determination of Infarct and Scar Size with TTC

Hearts were removed quickly and transferred to cold, isotonic 0.9% saline solution (Fresenius Kabi, Bad Homburg, Germany) supplemented with 1 mL heparin (5000 I.E.) (B. Braun, Melsungen, Germany). Subsequently, Evans blue dye solution (1% in PBS; Merck, Taufkirchen, Germany) was injected into the aorta through to the myocardium to delineate the ischemic AAR from the non-ischemic area. Stained hearts were stored at −20 °C for 1 h. Hearts were sliced (1 mm) in series along the long axis, weighed and incubated in TTC solution (1%) for 5–7 min at 37 °C to allow for the identification of viable and dead myocardium within the AAR. The infarct and viable area, as well as the AAR, were assessed by independent evaluators blinded to the experimental protocol using computer-assisted planimetry, taking the slice weights into account. The infarct area is expressed as a ratio of the dead area (TTC-negative) of AAR.

### 2.7. Mass Spectrometry

A pooled master mix of the respective lysates was used to generate a spectral library for the following experiments by data independent acquisition. Therefore, peptides dissolved in 10 mM ammonium acetate/0.4 mM formiate (pH 8.0) were separated on a C18 RP chromatography column. Peptides were fractionated at a flow rate of 12.5 μL/min with the following gradient with solvent B (84% (*v*/*v*) acetonitrile in 10 mM ammonium acetate, 0.4 mM formiate, pH 8.0): 3–10% for 10 min, 10–25% for 35 min, 25–40% for 20 min, 40–95% for 10 min, 95% for 5 min and 20 min equilibration at 3%. Fractionation was accomplished in 60 s intervals, concatenating each sample into 6 fractions.

For the generation of a spectral library, all lyophilized pH 8 derived fractions were dissolved in 0.1% (*v*/*v*) TFA with an appropriate amount of Biognosys Indexed Retention Time (iRT) kit (Bruker Daltonics; Part No:1816351) peptides and analyzed by nano LC-MS/MS using 1 μg, respectively. Samples were loaded on an Ultimate 3000 Rapid Separation Liquid chromatography (RSLC) nano system with a ProFlow flow control device coupled to a Q Exactive HF orbitrap mass spectrometer (both from Thermo Scientific, Darmstadt, Germany). Loaded peptides were concentrated on a trapping column (Acclaim C18 PepMap100, 100 μm, 2 cm) using 0.1% TFA at a flow rate of 10 μL/min. For sample separation, a reversed phase column (Acclaim C18 PepMap100, 75 μm 50 cm) using a binary gradient was used (3% solvent B (84% ACN with 0.1% TFA) for 10 min, a linear increase in solvent B to 35% for 90 min, a linear increase in solvent B to 95% for 10 min, followed by a linear decrease in solvent B to 3% for 5 min). MS survey scans were acquired using the following settings: a mass spectrometer was operated in data-dependent acquisition mode (DDA) with full MS scans from 300 to 1500 *m*/*z* at a resolution of 60,000. The automatic gain control (AGC) was set to 3 × 10^6^ with a maximum injection time of 120 ms. Most intense ions above a threshold ion count of 1.5 × 10^4^ were selected for fragmentation at a normalized collision energy (nCE) of 27%, following each survey scan. Dynamic exclusion was set to 15 s. Fragment ions were acquired at a resolution of 15,000 with an AGC of 5 × 10^4^ with a maximum injection time of 200 ms. Acquired data were imported into the software Spectronaut (Biognosys, version 14.9.201124.47784), and identification was accomplished using a mouse FASTA database selected from UniProt (www.uniprot.org). Processing settings were set as follows: the enzyme was trypsin; the minimum and maximum peptide lengths were set to 7 and 52, respectively; and missed cleavages were set to 2. Carbamidomethyl for cysteine was set as a fixed modification, and acetyl (Protein N-term) and the oxidation of methionine were set as variable modifications. All settings regarding the library generation including tolerances, identification, filters, iRT calibration and workflow were set to factory defaults.

For the data-independent acquisition (DIA) approach, the same nano LC-MS/MS setup as for the DDA acquisition was used. Full MS scans were acquired from 300 to 900 *m*/*z* at a resolution of 60,000. The automatic gain control (AGC) was set to 3 × 10^6^, and the maximum injection time was set to 20 ms. Full MS scans were followed by 15 DIA windows acquired at a resolution 30,000 with an AGC set to 3 × 10^6^ and nCE of 27%. For the analysis of the samples acquired with nano-LC-MS/MS in DIA mode, the data were introduced to the Spectronaut software and analyzed with a library-based search using the above-created spectral library. Search and extraction settings were kept as standard (BGS Factory settings).

### 2.8. Statistics

Statistical analysis was conducted using GraphPad Prism software (GraphPad Software, Version 10, Boston, MA, USA) and R (Version 4.1.2). Mass spectrometry data were presented as mean ± SD. Multiple comparisons were analyzed by paired one-way analysis of variance (ANOVA), followed by Tukey’s post hoc test, to identify group differences, or if they were not normal, a distributed Kruskal–Wallis test was performed. The correlation was calculated using Pearson’s correlation coefficient. Results with *p*-values ≤ 0.05 were considered statistically significant.

## 3. Results

**Figure 1:**
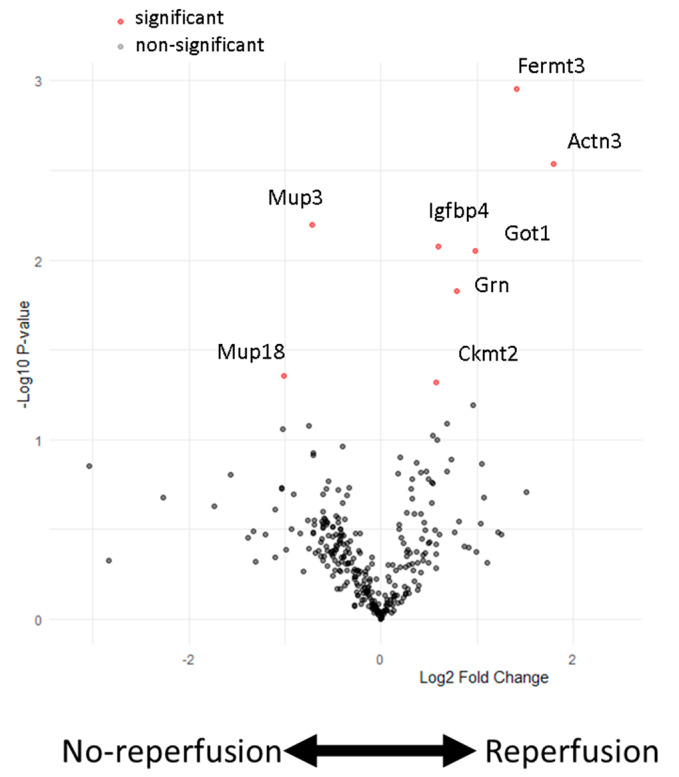
Protein abundance in the plasma of mice subjected to experimental AMI with and without reperfusion. A volcano plot illustrating the relative abundance (log2 fold changes) versus probability (−log10 *p*-values) in the plasma of mice that received either non-reperfused or reperfused AMI. Multiple *t*-test analyses with False Discovery Rate (FDR) correction were used to determine significance (indicated by red dots). Significant proteins are defined as those with a fold change > | ±1.5| and a *p*-value < 0.05. Two proteins are significantly upregulated in the non-reperfusion group (Mup3, Mup18), while six proteins are significantly upregulated in the reperfusion-operated mice (Ckmt2, Grn, Got1, Igfbp4, Got1, Actn3, Fermt3). *n* = 5 samples per group.

We examined the plasma protein abundance in two different murine models of AMI and their respective controls by untargeted mass spectrometry. The experimental groups included mice that were either untreated or sham-operated (open-chest surgery without coronary ligation) or received non-reperfused AMI (permanent ligation of the left anterior descending (LAD) coronary artery) or reperfused AMI (45 min transient LAD ligation followed by reperfusion). When comparing non-reperfused and reperfused AMI 24 h post induction, we identified eight differentially abundant proteins (Figure 1).

**Figure 2:**
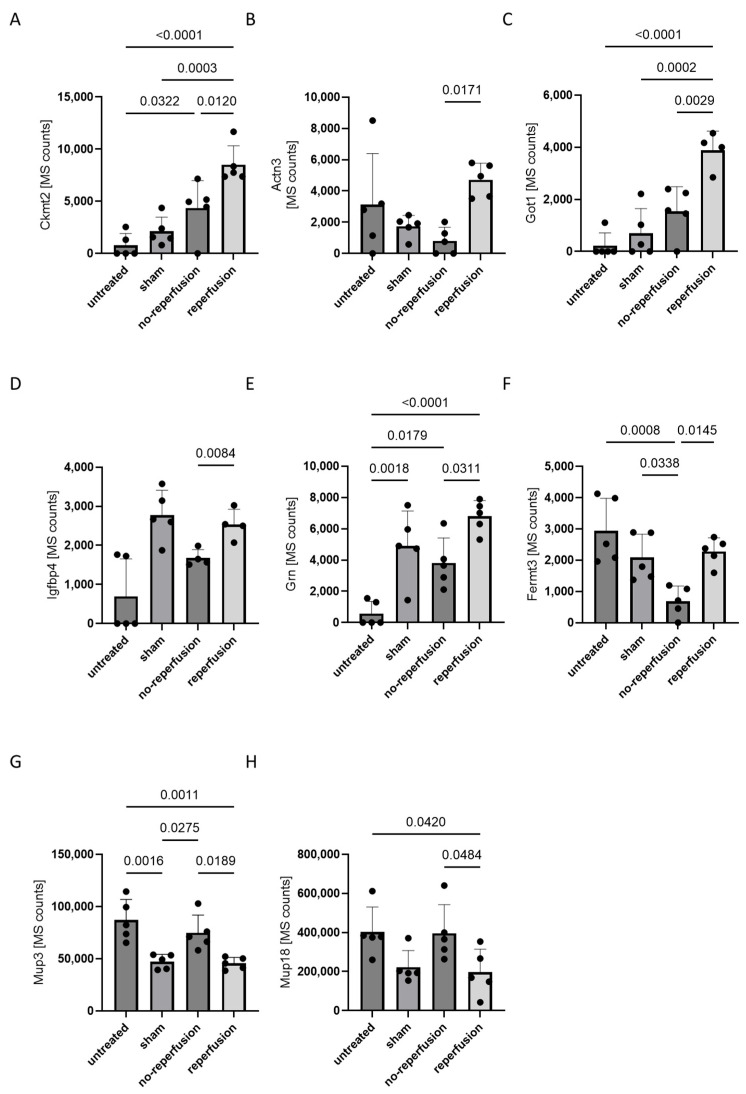
The mass spectrometric quantification of eight proteins significantly altered in plasma between non-reperfusion- and reperfusion-operated mice and their abundance in the respective controls. Plasma was isolated 24 h after the induction of sham-operated, non-reperfused AMI, and reperfused AMI and in untreated animals and was analyzed by untargeted mass spectrometry. Each bar plot represents the mean value with standard deviation (SD), with individual data points overlaid to show the distribution of measurements. Statistical significance between groups was determined using paired one-way ANOVA for normally distributed data or Kruskal–Wallis test for non-normally distributed data, with a *p*-value < 0.05 considered significant. Figure panels correspond to specific proteins: (**A**) Ckmt2, (**B**) Actn3, (**C**) Got1, (**D**) Igfbp4, (**E**) Grn, (**F**) Fermt3, (**G**) Mup3 and (**H**) Mup18. (**A**–**F**) show proteins that are upregulated in reperfusion-operated mice compared to non-reperfusion-operated mice, while (**G**,**H**) show proteins that are downregulated. Protein levels that were not detected are valued as 0. *n* = 5 per group.

Upregulated in the reperfused AMI group were fermitin family homolog 3 (Fermt3), alpha-actinin-3 (Actn3), insulin-like growth hactor binding protein 4 (Ifgbp4), glutamic-oxaloacetic transaminase 1 (Got1), granulin precursor (Grn) and mitochondrial creatinin kinese 2 (Ckmt2), while those elevated following non-reperfused AMI were Mup3 and Mup18. Although these proteins exhibited changes between non-reperfused AMI and reperfused AMI, only Ckmt2, Actn3 and Got1 were significantly upregulated compared to untreated, sham-operated and non-reperfused AMI animals (Figure 2A–C). Got1 was not detectable in all measured samples of the non-reperfusion and reperfusion groups compered to Ckmt2 and Tctn3, indicating its limitations in reproducibility. The other five proteins exhibited similar changes between the non-reperfused and reperfused AMI groups; however, they did not show significant changes compared to sham-operated animals (Figure 2D–H).

**Figure 3:**
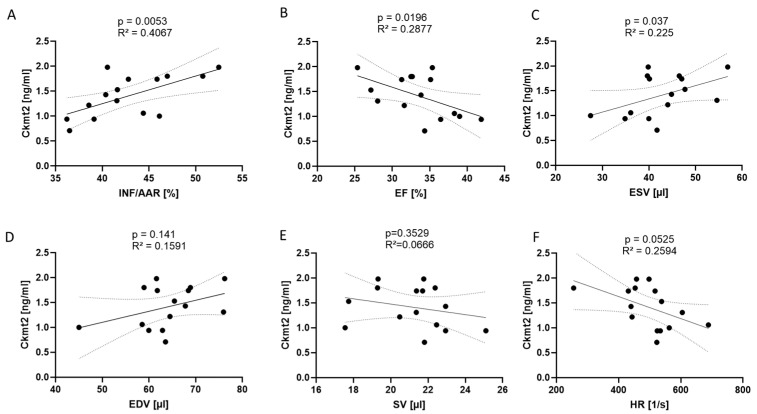
Ckmt2 correlates to infarct size and left ventricular dysfunction 24 h following reperfused myocardial infarction. (**A**) Ckmt2 plasma levels detected by ELISA show a significant positive correlation with infarct size (INF) relative to the area at risk (AAR), a negative relationship with (**B**) ejection fraction (EF) and a positive relationship with (**C**) end-systolic volume (ESV). (**D**) No significant correlation is observed between Ckmt2 levels and end-diastolic volume (EDV), (**E**) stroke volume (SV) or (**F**) heart rate (HR). Correlations are performed using Pearson’s correlation. Each dot represents an individual measurement from one mouse. Significance is defined as *p* < 0.05, and correlations are displayed with R^2^ values. *n* = 15.

To clarify whether rising levels of Ckmt2 were causally related to the specific effects of reperfusion damage (following 45 min of reperfusion after ischemia), we examined the relationship between these factors, infarct size and parameters of left ventricular (LV) function in a larger group of operated animals. We employed TTC staining to measure the extent of the infarct (INF) compared to the area at risk (AAR) 24 h after performing reperfused AMI. Concurrently, we evaluated LV function by echocardiography in the same animals. The analysis revealed significant associations between Ckmt2 levels in mouse plasma, as evaluated by ELISA, and infarct size determined by TTC staining (Figure 3A). Accordingly, Ckmt2 levels were negatively associated with ejection fraction (EF) 24 h post AMI induction while showing positive correlation with end-systolic volume (ESV) (Figure 3B,C), highlighting impaired systolic LV function in conjunction with larger infarct size. There was no significant correlation observed between end-diastolic volume (EDV), stroke volume (SV) and heart rate (HR) (Figure 3D–F). The simultaneous increase in Ckmt2 levels and higher ESV and a decline in EF indicates noticeable alterations in systolic heart function corresponding to the extent of tissue injury following an AMI.

## 4. Discussion

Despite significant progress in cardiovascular medicine, the mortality rate associated with AMI remains high. While reperfusion of the occluded area is essential to limit myocardial damage, it also introduces its own adverse effects [19,20]. Current pharmacological interventions, like ROS scavengers, have limited success in preventing reperfusion injury, emphasizing the need for a deeper understanding of its pathogenesis to develop more effective treatments [21,22]. The identification of reliable biomarkers for myocardial reperfusion injury is crucial for advancing treatment strategies. Over the past decades, multiple markers for AMI have been identified, including troponin, creatine kinase MB type (CK-MB), and Heart-Type Fatty Acid Binding Protein (hFABP) [23]. However, markers that are specific for myocardial reperfusion injury are limited [24,25]. Potential drivers of myocardial cell death during and after reperfusion include ferroptosis and mitochondrial membrane permeability [26]. While some markers are associated with ROS, new therapeutic targets for cardiac protection (e.g., SLC4A1) are under investigation [27,28]. To better understand the pathophysiology of cardiac reperfusion injury, it is crucial to identify and quantify suitable markers for this phenomenon.

Here, we aimed to identify plasma protein markers in murine experimental models of non-reperfusion and reperfusion, comparing their abundancy with those in untreated and sham-operated mice. Among the eight proteins that were significantly regulated 24 h post AMI (both models), only three showed significant upregulation in the plasma of mice with reperfused AMI compared to non-reperfused AMI, sham-operated, and untreated animals. This overlap indicates that these three proteins may serve as functional biomarkers for distinguishing reperfusion damage, with a reduced likelihood of being false positives.

Cytoplasmic aspartate aminotransferase (Got1) is associated with heart failure [29] and has been described as a potential marker for cancer [30]. Although Got1 shows significant upregulation in plasma of mice with reperfused AMI compared to non-reperfused AMI and sham-operated mice, its lower detection levels and inconsistent presence across samples suggest its limited reliability as a marker.

Alpha-actinin-3 (Actn3) is essential for the formation of actin and myosin filaments and sarcomeric structures. Specific mutations in this protein lead to decreased long-term survival in patients with chronic heart failure [31]. It is also described as a rapidly altering protein in the early response to myocardial infarction in pigs [32].

Mitochondrial proteins play a critical role in cellular energy production and are intricately linked to heart function [33]. Mitochondria undergo dynamic changes, such as increased calcium influx and oxidative stress, which contribute to the opening of the mitochondrial permeability transition pore. This leads to mitochondrial enlargement and the release of pro-apoptotic components into the cytoplasm, initiating the apoptotic cascade [6,7]. Ckmt2 is a pivotal enzyme within the mitochondrial creatine kinase system, which is essential for maintaining cellular energy homeostasis. In the heart, Ckmt2 is responsible for the rapid regeneration of ATP through the phosphocreatine shuttle, which is crucial during periods of high energy demand, such as during or after hypoxia [34]. The disruption of Ckmt2 activity can lead to impaired ATP production, exacerbating mitochondrial dysfunction and contributing to cell death during reperfusion injury [35]. Enhancing Ckmt2 expression or activity can protect against myocardial injury caused by ischemia-reperfusion injury [36]. In experimental models, the overexpression of mitochondrial creatine kinase has been shown to delay the opening of the mitochondrial permeability transition pore (mPTP), a key event in reperfusion-induced cell death. This delay reduces the extent of mitochondrial damage and, consequently, the overall infarct size [35,36]. The correlations observed between Ckmt2 levels and critical clinical outcomes, such as infarct size and left ventricular function, observed in our study underscore its possible value as an indicator of myocardial damage severity [35,36]. Unlike traditional biomarkers, which primarily reflect necrosis, Ckmt2 offers insights into the metabolic and mitochondrial health of cardiac cells, providing a more comprehensive assessment of myocardial injury.

Our study identified plasma protein markers in murine models of non-reperfusion and reperfusion, with Ckmt2 emerging as a particularly promising candidate. To advance Ckmt2 towards clinical application, further validation in clinical cohorts is necessary to establish its efficacy as a biomarker for acute myocardial infarction. Additionally, exploring its potential in guiding targeted therapies aimed at improving patient outcomes could significantly enhance its clinical utility. Beyond cardiovascular disease, investigating Ckmt2’s role in other mitochondria-related conditions, such as neurodegenerative diseases and certain cancers, may further broaden its relevance and inform future therapeutic strategies.

## 5. Conclusions

We identified Ckmt2 as a novel marker of reperfused but not non-reperfused AMI, correlating with infarct size and impaired cardiac function, highlighting its potential as a biomarker for reperfusion injury in acute myocardial infarction.

## Data Availability

Data are available via ProteomeXchange with identifier PXD055738.
